# Beyond Risk Factors: Spinal Epidural Abscess in a Young, Healthy Patient

**DOI:** 10.7759/cureus.74397

**Published:** 2024-11-25

**Authors:** Diana Guimarães, Mariana Silva, Sílvia Morgado, Lígia Reis, Paula Fernandes

**Affiliations:** 1 Anesthesiology, Unidade Local de Saúde do Alentejo Central, Évora, PRT

**Keywords:** asepsis, epidural abscess, neuraxial techniques, regional anesthesia, risk factors

## Abstract

Epidural abscess is a rare complication of neuraxial techniques, which, when left unnoticed, can lead to significant neurological deficits and poor outcomes. Identification of patients at high risk and the conduct of a strict aseptic technique are some of the measures that play an important role in epidural abscess prevention. Prompt recognition and treatment of epidural abscesses are essential. Close monitoring in the perioperative period for new-onset clinical signs and symptoms is of major importance. When an epidural abscess is suspected, early laboratory assessment and magnetic resonance imaging (MRI) are indicated. Treatment options include early empiric and posteriorly antibiogram-guided antibiotic therapy and surgical decompression of the epidural abscess.

This case presents a young male patient with no significant medical history or predisposing risk factors who developed an epidural abscess following epidural catheter placement for osteosynthesis of a left tibial plateau fracture. The patient's absence of additional predisposing conditions, besides two attempts at epidural catheter placement, underscores the complexity and unpredictability of such infections.

## Introduction

Epidural abscesses are rare but serious complications associated with epidural anesthesia or analgesia, characterized by purulent collections in the epidural space. These infections can result in spinal cord compression, leading to significant neurological deficits.

Risk factors include repeated epidural catheterization, prolonged catheter use, pre-existing infections, diabetes, and immunosuppression [1,2]. Since most of the predisposing factors allow for the invasion of the epidural space by skin flora, *Staphylococcus aureus* is involved in two-thirds of spinal epidural abscess (SEA) cases [1].

Symptoms can progress from back pain at the puncture level and nerve root radiating pain to motor and sensory deficits, as well as bladder and bowel dysfunction and, ultimately, paralysis [1]. Back pain, fever, and neurological deficits compose the traditional triad of the most common symptoms in SEA, although only a minority of patients present with it [1].

The diagnosis of SEA is based on clinical findings and supported by laboratory and imaging data, namely, blood cultures and magnetic resonance imaging (MRI), respectively, but drainage is necessary for diagnostic confirmation [1]. Nonspecific findings such as leukocytosis and elevated erythrocyte sedimentation rate and C-reactive protein (CRP) might be the only analytical features on presentation, leading to misdiagnosis [1,2]. As a result, a high suspicion level and awareness for prompt recognition of a SEA is necessary, particularly in a patient with risk factors.

As far as treatment is concerned, systemic antibiotics combined with expeditious decompressive laminectomy and debridement of infected tissues are indicated [1,2]. While awaiting blood culture results, empirical antibiotic therapy should be initiated and, posteriorly, guided by the antibiogram and continued for at least six weeks [1].

This case highlights the need for a high index of suspicion for infectious complications in cases of repeated epidural catheterization, even in the absence of traditional risk factors, and underscores the role of MRI as the preferred imaging modality for early diagnosis in similar clinical settings.

## Case presentation

A 41-year-old male patient, classified as American Society of Anesthesiologists (ASA) physical status 1 and with no significant past medical history, was admitted to the orthopedics department for osteosynthesis of a left tibial plateau fracture. The surgery was performed under combined spinal-epidural (CSE) anesthesia. The technique was conducted under strict aseptic conditions (cap, mask, sterile gown, and gloves) with skin disinfection using an alcohol-based solution (Cutasept® F, HARTMANN, Heidenheim, Germany). A CSE kit, which included an 18G Tuohy needle and a 27G spinal anesthesia needle, was used. Two grams of cefazolin were administered preoperatively for surgical antibiotic prophylaxis. The surgery proceeded uneventfully, and postoperative analgesia was managed with an epidural disposable elastomeric infusion pump system (Easypump®, B. Braun, Melsungen, Germany) containing 0.15% levobupivacaine, infused at 5 mL/hour.

On the first postoperative day, the anesthesiology team was alerted to inadequate pain control. Upon examination, it was confirmed that the epidural catheter had dislodged from the epidural space. The puncture site was dry, with no signs of inflammation, bleeding, or cerebrospinal fluid (CSF) leakage. A peripheral nerve block was considered but could not be performed due to the immobilization of the limb. Consequently, a second epidural block was performed in the operating room under the same aseptic technique. The procedure was successful, and postoperative analgesia was effectively managed with epidural administration of 0.15% levobupivacaine using a new disposable elastomeric infusion pump system for an additional 48 hours. Pain control was adequate throughout the remainder of the hospitalization, and the patient experienced no adverse effects from the medication, namely, any neurological deficits. The epidural catheter was removed without incident, and the patient was discharged on postoperative day 3.

**Figure 1:**
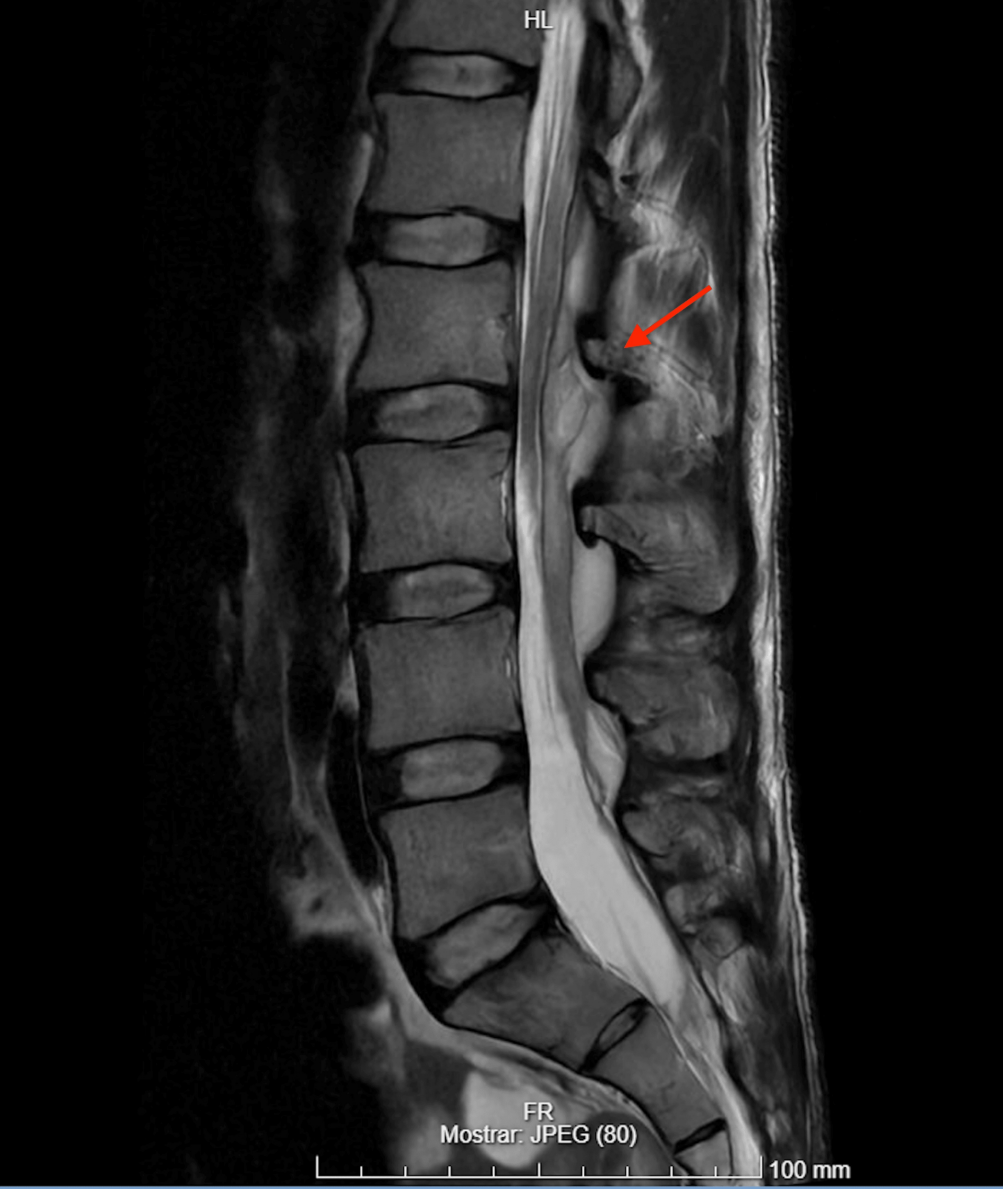
MRI revealing an epidural abscess (red arrow) extending from L1 to L4 with paravertebral involvement at the L2-L3 level MRI: magnetic resonance imaging

Five days after discharge, the patient presented to the emergency department (ED) with poorly controlled lower back pain but no other significant symptoms. A physical examination and a lumbar computed tomography (CT) scan revealed no abnormalities, and the patient was discharged with optimized analgesic therapy. However, two days later, on postoperative day 7, he returned to the ED with severe, localized pain at the lumbar puncture site, now accompanied by cervicalgia. Neurological examination was unremarkable, with no sensory or motor deficits, no sphincter dysfunction, no neck stiffness, and preserved symmetrical reflexes. The puncture site showed no signs of blood or CSF leakage, except for a pustule. Laboratory tests revealed elevated inflammatory markers, namely, leukocytosis and neutrophilia, with a leukocyte count of 18,000/μL, neutrophils at 88%, and C-reactive protein (CRP) at 18.3 mg/dL. A repeat lumbar CT scan demonstrated a new posterior epidural collection at the L2-L3 level, causing minimal compression of the dural sac. This finding had not been evident on the prior scan. An MRI confirmed a heterogeneous epidural collection extending from L1 to L4 with paravertebral involvement at the L2-L3 level, consistent with an epidural abscess (Figure 1).

The patient underwent urgent surgical decompression and drainage of the epidural abscess at L2-L3.

Intraoperative cultures were positive for methicillin-sensitive *Staphylococcus aureus* (MSSA). Post-surgical antibiotic therapy was tailored to the antibiogram, consisting of trimethoprim-sulfamethoxazole and clindamycin. The patient's postoperative course was smooth, and he was discharged on postoperative day 11, having completed a six-week course of antibiotic therapy. At follow-up, the patient showed full recovery, with no neurological deficits and complete resolution of symptoms.

## Discussion

It is estimated that the incidence of major complications of central neuraxial anesthesia in non-obstetric patients may range from 1:6000 to as high as 1:1000 epidural procedures [3]. Although SEA are rare, their incidence may be increasing largely due to the growing prevalence of patients at risk for its development [2]. The development of an epidural abscess in a young, otherwise healthy patient, without classical predisposing factors beyond undergoing two epidural blocks, is a notably rare event. Typical risk factors for SEA include diabetes mellitus, immunosuppression, malignancies, advanced age, or prior invasive spinal procedures [1,2]. In this case, the absence of these traditional risk factors highlights the unpredictability of abscess development following neuraxial anesthesia. While hematogenous dissemination or local spread from adjacent tissues are known pathways for infection to reach the epidural space, no clear distant source of infection was identified in this case [1,2]. However, the performance of two attempts at epidural catheter placement is debatable and might have played an important role in the case.

The most common etiological agent associated with SEAs is *Staphylococcus aureus*, particularly methicillin-resistant *Staphylococcus aureus* (MRSA). In this case, cultures were positive for MSSA. Other potential pathogens include *Staphylococcus epidermidis*, *Escherichia coli*, and *Pseudomonas aeruginosa* [1,2].

Diagnosing a SEA is challenging due to its nonspecific clinical presentation and the absence of the classic triad, namely, back pain, fever, and neurological deficits, in most cases. Fewer than 20% of patients present with all three components initially, making early detection difficult [1,3]. In this case, the patient's primary complaints of lower back pain and cervicalgia align with the most prevalent symptom of SEA, which occurs in up to 75% of cases. [1]. This pain is typically focal and progressive, often preceding the onset of neurological deficits, which may range from radiculopathy to motor or sensory impairments. Additionally, the diagnosis of SEA is complicated by the delayed onset of symptoms, which frequently emerge after the epidural catheter is removed, often after the patient has been discharged from the hospital [3].

The absence of fever and neurological deficits at presentation is consistent with previous reports that fever is often absent, contributing to delayed diagnosis. Laboratory findings of elevated inflammatory markers (leukocytosis, C-reactive protein, and erythrocyte sedimentation rate) are common but nonspecific. In this case, the patient exhibited elevated inflammatory markers and blood cultures, which are positive in 40%-60% of SEA cases, later confirmed methicillin-sensitive *Staphylococcus aureus* as the causative organism [1,2].

Imaging is crucial for diagnosis. The initial CT scan of the lumbar spine showed no abnormalities, which is not uncommon, as CT has limited sensitivity for early or soft tissue changes related to SEA [1,2]. An MRI with contrast, the gold standard for detecting epidural abscesses due to its superior sensitivity for soft tissue evaluation, revealed a heterogeneous epidural collection at L2-L3, extending to L1-L4. This imaging was critical for confirming the diagnosis and guiding timely surgical intervention. In this case, considering the prior history of two punctures for epidural catheter placement and clinical signs suggestive of infection, MRI should have been chosen as the initial imaging study over a CT scan.

Preventive measures during central neuraxial blockade (CNB) play an essential role in reducing infection risk. Therefore, the removal of jewelry followed by thorough handwash with a surgical scrub solution and the use of a cap, mask, and sterile gloves as well as a large sterile drape are recommended both for spinal and epidural techniques [4,5] whether for single-shot or catheter placement purposes, although there is insufficient literature to evaluate differences in infectious complications between them [5]. On the need to use sterile gowns in these techniques, there is, however, some controversy [4,5].

The choice of antiseptic for skin preparation before CNB is another critical factor. There is a certain controversy between chlorhexidine and povidone-iodine, which are available both in aqueous and alcoholic forms. However, chlorhexidine has shown superiority when compared to povidone-iodine [5] due to its broader antiseptic spectrum, faster onset, longer duration of action, lower incidence of skin reactions and catheter colonization, and preservation of action even in the presence of blood [4]. Portuguese national guidelines on regional anesthesia also highlight chlorhexidine as a preferential antiseptic [6]. Also, an alcoholic chlorhexidine solution seems to be associated with less bacterial growth when compared to its aqueous equivalent. However, care must be taken with chlorhexidine due to its potential neurotoxicity, so it must be kept away from CNB equipment, and the technique must only be initiated once the solution has fully dried on the skin to prevent neuraxial contamination [4-6]. Therefore, in terms of chlorhexidine concentration, the 0.5% alcoholic solution shows the safest balance between the risk of infection and the risk of neurotoxicity when compared to a 2% alcoholic solution [4]. In the present case, an alcoholic solution composed of isopropanol and benzalkonium chloride was used for skin preparation before CNB. The use of a less ideal skin antiseptic, such as Cutasept®, compared to agents such as chlorhexidine, might have contributed to a higher risk of contamination during the procedure.

Other preventive measures include the identification of patients at risk for infectious complications through a thorough medical history, physical examination, and review of laboratory studies and subsequent consideration of alternatives to neuraxial techniques [5].

Single-use antiseptic packets and occlusive dressings at the catheter insertion site should be employed. Moreover, unnecessary disconnections of neuraxial delivery systems should be minimized, unwitnessed accidentally displaced catheters should be removed, and catheters should be promptly discontinued when no longer clinically necessary [5].

Given the rarity and severe consequences of spinal epidural infections, prospective, randomized clinical trials to determine optimal treatment are both impractical and ethically challenging. However, most retrospective studies agree that the preferred approach is surgical drainage combined with systemic antibiotics [1-3]. The patient's preoperative neurological status is the strongest predictor of the final outcome, and because the progression of neurological impairment can be unpredictable, early decompressive laminectomy and debridement are often necessary [1,3]. The decision to proceed with surgery depends on the severity of symptoms, abscess size and location, risk of spinal instability, and the patient's overall health and comorbidities [1,7]. In patients with smaller or less symptomatic SEAs, medical management with intravenous antibiotics may be an option [7].

Empirical antibiotic therapy should be initiated after obtaining blood cultures, with broad-spectrum coverage such as vancomycin for MRSA and ceftriaxone for gram-negative bacteria [1]. Once culture results are available, the antibiotic regimen should be tailored to the identified pathogens. In this case, the patient's therapy was adjusted to clindamycin and trimethoprim-sulfamethoxazole, ensuring targeted treatment of the causative organism. Antibiotic treatment typically lasts six weeks, depending on the clinical response and any underlying conditions [1].

## Conclusions

In conclusion, this case highlights the critical need for early recognition and intervention in managing SEAs, particularly in patients lacking classical risk factors. The development of SEAs following epidural anesthesia can result in significant morbidity if not promptly diagnosed and treated. Early MRI, combined with clinical and laboratory assessments, is essential for preventing irreversible neurological damage. This case underscores the importance of adhering to strict aseptic protocols and vigilant monitoring during the perioperative period.

With the anticipated NAP8 report: Complications of Regional Anaesthesia, coordinated by the Royal College of Anaesthetists, forthcoming insights into neuraxial anesthesia complications will likely enhance clinical practice and awareness, reducing the risk of severe outcomes from rare complications such as SEA.
